# Distribution and Dietary Predictors of Urinary Phthalate Metabolites among Pregnant Women in Shanghai, China

**DOI:** 10.3390/ijerph16081366

**Published:** 2019-04-16

**Authors:** Xin He, Jiajie Zang, Ping Liao, Yang Zheng, Ye Lu, Zhenni Zhu, Yan Shi, Wenjing Wang

**Affiliations:** 1 Laboratory of Functional Medicine, Division of Chronic Non-Communicable Diseases and Injury, Shanghai Municipal Center for Disease Control and Prevention, Shanghai 200336, China; hexin@scdc.sh.cn (X.H.); liaoping@scdc.sh.cn (P.L.); luye@scdc.sh.cn (Y.L.); 2 Department of Nutrition Hygiene, Division of Health Risk Factor Monitoring and Control, Shanghai Municipal Center for Disease Control and Prevention, Shanghai 200336, China; zangjiajie@scdc.sh.cn (J.Z.); zhuzhenni@scdc.sh.cn (Z.Z.); 3 Department of NCD surveillance, Division of Chronic Non-Communicable Diseases and Injury, Shanghai Municipal Center for Disease Control and Prevention, Shanghai 200336, China; zhengyang@scdc.sh.cn

**Keywords:** phthalate metabolites, pregnancy, dietary, edible seaweed

## Abstract

The exposure of pregnant women to phthalates is a major concern due to their adverse effect on developmental outcomes. Diet is an important pathway for exposure to phthalate compounds. Nevertheless, studies on dietary exposure of pregnant women to phthalates in China are limited. We aimed to assess the distribution and dietary predictors of phthalate exposure among pregnant women in China. We measured the levels of 10 urinary phthalate metabolites using high-performance liquid chromatography coupled with tandem mass spectrometry in 210 pregnant women as part of the 2015 China National Chronic Disease and Nutrition Survey in Shanghai. We assessed the urinary specific gravity-adjusted phthalate metabolite levels along with potential demographic and dietary predictors. Multivariable linear regression analysis was used to examine the relationship between each potential demographic variable and dietary predictor and urinary phthalate metabolites. Seven urinary phthalate metabolites were detected in >95% of pregnant women. The geometric mean (GM) of urinary phthalate biomarker values were highest for monobutyl phthalate (GM: 25.29 ng/mL) and monoisobutyl phthalate (GM:11.18 ng/mL). Multivariate regression analysis indicated that a lower educational level was associated with elevated urinary phthalate metabolite levels. Edible seaweed consumption had a positive correlation with urinary monoethyl phthalate and monoisobutyl phthalate levels, and the total molar sum of Di-(2-ethylhexyl) phthalate metabolites. These findings offer important data on the dietary exposure to phthalates in pregnant Chinese women and suggest interventions to improve food safety.

## 1. Introduction

Phthalates (phthalic acid esters, PAEs) are well-known chemicals disrupting the endocrine system and are abundantly produced worldwide, given their multiple uses as plasticizers or solvents in food packaging, personal care products, and other domestic products [1,2]. Phthalates are found in various parts of the environment, including water, indoor dust, sediments, and organisms [3,4,5,6]. Exposure to these chemicals may occur through digestion ingestion, skin absorption, or inhalation in daily life. In the body, phthalates are metabolized into their respective monoester metabolites via hydrolysis and oxidation, are subsequently conjugated with glucuronide, and then mostly excreted in urine within 24 h [7,8]. Thus, human bio-monitoring of urinary phthalate metabolites can be used as a sensitive, specific, and reliable method for measuring phthalate exposure [9].

Epidemiological and animal studies have shown that the main adverse health effects of phthalate exposure include increased blood pressure [10], impairment of thyroid and cardiovascular activity [8,11], reproductive and developmental toxicity [12,13], and allergic diseases such as asthma and allergy in children [14,15]. Previous studies have found that phthalates can penetrate the placenta and may affect the fetus [16]. Exposure to phthalates in utero may have adverse health effects on children, including poor birth outcomes [17], impaired neurodevelopment, and behavioral syndromes in childhood [2,18,19]. Therefore, considerable attention should be paid to phthalate exposure in pregnant women.

Phthalates can migrate into food through the production, packaging, storage, and transport of food, and hence, diet is considered a significant exposure route for phthalates [20,21]. The parentalcompounds of monobutyl phthalate (MnBP), monoisobutylphthalate (MiBP), monobenzyl phthalate (MBzP), and mono(2-ethylhexyl) phthalate (MEHP) were frequently detected in various daily foods in food monitoring surveys [22]. Epidemiological studies in the general population suggest that certain food groups, such as poultry, seafood, milk, dairy products, meat, eggs, and egg products have a close relationship with anelevated phthalate body burden in different countries [23,24,25,26]. However, other studies have shown contrasting evidence that egg and milk consumption is related to lower urinary phthalate metabolite levels [27,28]. Previous studies on the association between dietary intake and phthalate concentration among pregnant women have been performed in the US and Netherlands [28,29,30]. A limited number of bio-monitoring studies have been conducted on the relationship between diet intake and phthalates distribution in Chinese pregnant women across all trimesters. Therefore, in the present study, we aimed to assess the distribution of phthalate exposure in pregnant women to evaluate whether phthalate exposure is associated with the increased intake of certain types of food based on Chinese dietary habits. These findings could help offer specific interventions to reduce the high risk of dietary phthalate exposure during pregnancy. 

## 2. Methods and Materials

### 2.1. Study Population

Subjects were enrolled from the 2015 China National Chronic Disease and Nutrition Survey, including 210 pregnant women recruited from 7 survey centers in Shanghai. Each survey center enrolled 30 pregnant women, and the subjects were equally divided across trimesters. All pregnant women were residents of Shanghai for >6 months. They provided blood and urine samples and completed physical examinations and face-to-face questionnaire interviews on theirdemographic and diet information. The study was approved by the Ethical Committee of the National Institute of Nutrition and Health, and National Institute of Chronic Non-Communicable Diseases, Chinese Center for Disease Control and Prevention (CDC). All study participants provided written informed consent.

### 2.2. Socio-Demographic Characteristics

The participants’ socio-demographic data, including maternal age, race, gestational week, pre-pregnancy weight, location, highest education level, occupation, annual household income, andhealth habits (such as cigarette smokingand alcohol consumption status), were obtained using a standard self-reported questionnaire. The subjects’ height on standing was measured using an automatic instrument. The pre-pregnancy body mass index (BMI) was calculated by dividing the self-reported pre-pregnancy weight(kg)by the squareof height (m) (BMI = weight [kg]/height squared [m^2^]).

The gestational week was categorized into the first (0–12 weeks), second (13–27 weeks), and third trimester (28–40 weeks). Based on the pre-pregnancy BMI, subjects were categorized as underweight (BMI < 18.5), normal (BMI: 18.5–24.9), or overweight/obese (BMI ≥ 25). Current cigarette smoking and alcohol consumption (liquor/beer/wine) habits within 30 days of the investigation date were recorded. Previous cigarette smoking and alcohol consumption habits 30 days before the investigation date were also recorded. 

### 2.3. Dietary Questionnaires

A standard food frequency questionnaire (FFQ) of the Chinese CDC was used to assess dietary intake [31]. In total, 210 participants completed the FFQ to record the frequency and intake of particular food items consumed in the previous 6 months (never, daily, weekly, and monthly). The FFQ included a total number of 55 food items, categorized into 12 groups, such as staple food, beans, vegetables, edible mushrooms and algal food, fruits, milk products, meat, seafood, egg products, beverages, alcohol, and others. 

### 2.4. Urine Collection and Analysis

Spot urine samples were collected in a 50 mL polypropylene (PP) tubes from each participant between 7:30 and 9:00. Urine samples were aliquoted in four 5 mL PP tubes and were frozen at −20°C. Ten phthalate metabolites, including monobutyl phthalate (MnBP), monoisobutyl phthalate (MiBP), monoethyl phthalate (MEP), mono(2-ethyl-5-carboxypentyl) phthalate (MECPP), mono(2-ethylhexyl) phthalate (MEHP), mono(2-ethyl-5-oxohexyl) phthalate (MEOHP), mono(2-ethyl-5-hydroxyhexyl) phthalate (MEHHP), monomethyl phthalate (MMP), monobenzyl phthalate (MBzP), and mono(3-carboxypropyl) phthalate (MCPP), were measured using high-performance liquid chromatography coupled with tandem mass spectrometry (HPLC-MS/MS) (Agilent 1290, Agilent 6495, Agilent Technologies Co., California, USA) according to the manufacturer’s instructions [32]. The corresponding ^13^C4-isotope-labeled analogs of phthalate metabolites were used as internal standards. All external and internal standards were obtained from Toronto Research Chemicals (Ontario, Canada) and CDN Isotopes (Quebec, Canada).

Urinary dilution was corrected using specific gravity (SG). Several studies indicated that, for pregnant women, urinary SG adjustment may be better than creatinine adjustment [33], as the creatinine concentration may be high or low during pregnancy [34,35]. Urinary SG was measured using a urine Urit-500B analyzer (Guilin Urit Electronics Group Co. Guangxi, China).Individual metabolite was adjusted for SG, using the formula: Pc = P[(SGp−1)/(SGi−1)], where Pc is the SG-corrected phthalate metabolite concentration (ng/mL), P is the measured urinary phthalate metabolite concentration, SGp is the median urinary SG level, and SGp is the measured individual urinary SG [36]. 

### 2.5. Statistical Analysis

SPSS software (Version19.0) was used for data analysis. Demographic characteristics were expressed as mean ± standard deviation (SD) or percentage (%). As the concentrations of phthalate metabolites were positively skewed, the geometric mean (GM) and 95% confidence interval (CI) values were used to illustrate the distributions of both unadjusted and SG-adjusted concentrations of phthalate metabolites. As MEHP, MEOHP, MEHHP, and MECPP share a common parent compound, di-(2-ethylhexyl) phthalate (DEHP), the molar sum of the concentrations of the DEHP metabolites (ΣDEHP) was recorded, using the following formula: ΣDEHP = (MEHP/278.34) + (MEOHP/292.33) + (MEHHP/294.34) + (MECPP/308.33), in nmol/L [37].

We considered 13 food items as potential dietary predictors of urinary phthalate metabolites. Among these, pickles, edible seaweed, and tofu were specific to Chinese dietary habits, whereas the others considered were predictors of phthalate exposure reported in previous studies [22,23,24,25,26]. Maternal demographic variables, such as maternal age, gestational age, location, education level, pre-pregnancy BMI, and occupation, were recorded, given their role as important demographic adjustment factors in pregnancy studies and phthalate exposure studies. Smoking and drinking status were not considered because >96% participants were never smokers or alcohol drinkers. Potential predictors were dichotomized based on approximate median values or inherent groupings. Univariate comparisons among maternal demographic characteristics and dietary predictors were performed using analysis of variance (ANOVA) or Student’s *t*-test. A *p* value of <0.05 was considered statistically significant. If the variables were considered significant on univariate analysis, multivariable linear regression analysis was used to determine whether a relationship between potential demographic characteristics, dietary predictors, and urinary phthalate biomarkers was present.

## 3. Results

Table 1 indicates the main demographic characteristics of the research population. The mean maternal age was 29.02 years (range, 20.0–39.0 years). Participants were evenly grouped across the trimesters, and the mean gestational week was 20.43 weeks. The subjects had lived in Shanghai for >6 months, and 42.9% had resided in an urban area. Approximately 16.7% of the subjects were overweight (BMI: 25.0–27.9 kg/m^2^) and obese (BMI ≥ 28.0 kg/m^2^) before pregnancy, whereas 10% were underweight (BMI < 18.5 kg/m^2^) before pregnancy. Almost 38.5% of the study population had completed university and higher education. The proportion of subjects who worked in production (farmers/factory workers), services, management/technician, and housework fields were 4.8%, 14.8%, 47.6%, and 25.2%,respectively. Moreover, >98% were never smokers and >96% were never alcohol drinkers.

The distributions of the 10 phthalate metabolites are shown in Table 2. The detectable frequencies ranged from 12.3% to 100%. MnBP, MiBP, and MEHP were detected in almost 100% of the subjects. MEP, MECPP, MEOHP, and MEHHP were detected in >95% of the samples. The other 3 phthalate metabolites (MMP, MCPP, and MBzP) with low occurrence (<50% detection) were excluded from further analysis. Among the phthalate metabolites analyzed in this study, MnBP was the metabolite with the highest GM (25.29 ng/mL), followed by MiBP (GM: 11.18 ng/mL), MEP (GM: 6.33 ng/mL), MECPP (GM: 5.19 ng/mL), MEOHP (GM: 3.00 ng/mL), MEHHP (GM: 1.73 ng/mL) and MEHP (GM: 1.36 ng/mL).The SG-adjusted concentrations of phthalate metabolites were similar to the SG-unadjusted concentrations. The limits of detection for the urinary levels of the 10 phthalate metabolites were 0.1–2.0 ng/mL.

Univariate analyses were used to confirm whether urinary phthalate biomarkers were associated with maternal demographic characteristics or certain potential predictors. ANOVA of SG-adjusted phthalate metabolites, classified based on the maternal characteristics, indicated that the GM concentration of MnBP was higher in pregnant women residing in suburban areas (*p* = 0.011), as well as in those with lower pre-pregnant BMI (<18.5; *p* = 0.028) and lower education level (less than high school; *p* < 0.01). Moreover, the GM concentration of MEP gradually decreased with increasing gestational week (*p* = 0.018), and the urinary MiBP concentration was higher in pregnant women who were younger (age: 20–24 years; *p* = 0.021), underweight before pregnancy (pre-pregnant BMI < 18.5; *p* = 0.009), and with a lower education level (less than high school; *p* = 0.001). The levels of other phthalate metabolites were not significantly associated with maternal age, location, pre-pregnancy BMI, maternal education, and employment status in our cohort (Table 3). In a bivariate analysis of dietary factors, several food groups were found to be superior potential predictors of urinary phthalate metabolite levels. We found a significant positive correlation between edible seaweed and MEP (*p* = 0.008) and MiBP (*p* = 0.035) levels, as well as a significant inverse correlation between milk consumption and MnBP (*p* = 0.023) and MiBP (*p* = 0.043) levels. Participants who consumed more pork (*p* = 0.020) and marine fish (*p* = 0.042) had lower MEP levels (Table 4).

## 4. Discussion

In the present cross-sectional study, we identified 10 urinary phthalate metabolites, and observed the widespread exposure to these phthalates in our study population. Similarly, a Chinese study conducted in 2010 found that 14 phthalate metabolites were ubiquitous in spot urine samples [38]. MnBP was the most common metabolite detected in our participants, followed by MiBP, MEP, MECPP, MEOHP, MEHHP, and MEHP. The pattern of phthalate exposure in the present study was similar to that noted in previous studies of pregnant women in Mainland China [39,40]. However, compared with the women in the NHANES 2011–2012 study, as well as the recent study of pregnant women in Charleston, US, the GM of MEP levels in the present study (6.33 ng/mL) was almost six- to eight-fold lower (37.7 ng/mL and 47 ng/mL, respectively) [30,41]. In contrast, the MnBP concentration was two-fold higher in the present study as compared to those reported in the US (GM: 25.29 ng/mL vs.7.14 ng/mL and 13.7 ng/mL, respectively) [30,41]. Furthermore, MBzP was not commonly detected in pregnant Chinese women, with detection frequencies of 45.8% in the present study, 49.3% in the Ma’anshan Birth Cohort [39], 56.7% in a Taiwan birth cohort study [42] and 74.8% in the Wuhan pregnant women study [43]. However, the detection frequencies of maternal urinary MBzP were >95% in the MIREC cohort from Canada in 2008–2011 [44], the UK DEMOCOPHES pilot study conducted in Europe [45], and the US NHANES 2011–2012 [41]. These different levels and detection frequencies may reflect the different patterns of use of the parent phthalate compounds in these countries. 

We found that the demographic predictors of increased phthalate burden included lower education level, similar to that noted in other pregnant women cohorts. For example, a cohort in the Netherlands indicated that younger age, lower educational level, and lower income level were positively associated with MBP and MBzP levels [46]. Similarly, in a Spanish cohort, lower education level exhibited a relationship with higher ∑DEHP and MEP concentrations [47]. Moreover, the Charleston SC cohort indicated that college-educated women had the lowest phthalate concentrations (MBP, MBzP, MiBP, MEP, and MMP), as compared to less educated pregnant women [30]. The lack of awareness of plasticizer contamination was a potential reason explaining the elevated phthalate concentrations in less-educated pregnant women. Recently, certain studies indicated that using inexpensive personal care products made with large amounts of phthalates and residing in older houses may be related to the increased phthalate concentrations in low-income populations [42,48,49]. Many of the subjects did not report their income level in the present study, but the education level could reflect the income level to a certain extent [50], and hence, we may conclude that elevated phthalate exposure could be more likely in a less-educated population.

The major finding of the present study was the positive association between edible seaweed intake and urinary MEP, MiBP and ∑DEHP levels, after adjusting for covariates. To our knowledge, no other study has reported this result. A food monitoring study in China showed that among 78 representative samples of widely consumed foods, the content of phthalates is greatest in seafood [51]. Epidemiological studies also reported that seafood may have positive associations with urinary MBP concentrations [21]. However, it is unclear whether edible seaweed was included in the seafood category in these studies. Edible seaweed is a type of marine red algae and is popular in many Asian countries. However, the persistence of phthalates in the aquatic environment may inevitably lead to bioaccumulation of phthalate pollutants in edible seaweed [7,52]. The introduction of phthalates into seaweed may also occur during production and packaging. Furthermore, a study found that red algae can synthesize DEHP and DnBP de novo [53]. In that study, Chen found that red algae could synthesize 2 phthalate esters (DEHP and DnBP); however, it was unclear whether red algae could also synthesize other phthalate esters de novo [53]. Further research, including basic studies, food monitoring surveys, and intervention studies are needed, since the origin of such phthalate contamination in edible seaweed remains unclear.

A major feature of the present study is that it is the first study to evaluate the dietary exposure to phthalates among pregnant women in Shanghai across all three trimesters and can hence provide important data for evaluating the overall dietary exposure of pregnant Chinese women to phthalates. Moreover, given the difference between Chinese and Western diet patterns, we focused on the unique Chinese diet consumption characteristics, which could provide options for improving food safety in the sensitive pregnant women. The present study also had certain limitations. First, this was a cross-sectional study, and hence cannot provide direct evidence to indicate that dietary intake is a cause of urinary phthalate metabolites. Second, it is unclear whether single spot urine levels can reflect prenatal exposure to phthalates over a long time, as they usually have a short half-life. Third, the other possible routes of human exposure to phthalates, such as housing characteristics and personal care products, were not considered as covariates in the present study [42,48].

## 5. Conclusions

In conclusion, the present study showed that phthalate exposure is prevalent among pregnant women in Shanghai, with MnBP and MiBP being the most common phthalate metabolites detected. We observed that certain maternal demographic characteristics were associated with elevated phthalate metabolite concentrations, including a lower educational level. Edible seaweed consumption may be a dietary predictor of elevated MEP, MiBP, and ∑DEHP concentrations among pregnant women, which suggests that future analyses should carefully consider this factor.

## Figures and Tables

**Table 1:** Characteristics of the participating pregnant women.

Maternal Characteristics	N = 210	Mean ± SD or Percentage (%)
Age at delivery (years)		29.02 ± 3.96
20~24	23	11.0%
25~29	108	51.4%
30~34	54	25.7%
35~40	25	11.9%
Gestational age		20.43 ± 5.06
First trimester	70	33.3%
Second trimester	70	33.3%
Third trimester	70	33.3%
Location		
Urban	90	42.9%
Suburban	120	57.1%
Pre-pregnancy BMI		21.55 ± 3.29
Underweight (<18.5)	21	10.0%
Normal (18.5–24.9)	154	73.3%
Overweight/Obese (≥25)	35	16.7%
Education level		
High school or less	73	34.8%
College	56	26.7%
University or higher	81	38.5%
Occupation		
Production	10	4.8%
Service	31	14.8%
Management and technician	100	47.6%
Housework	53	25.2%
Others	16	7.6%
Cigarette smoking		
Current smoker	0	0%
Former smoker	3	1.4%
Never smoker	207	98.6%
Alcohol consumption		
Current drinker	4	1.9%
Former drinker	4	1.9%
Never drinker	202	96.2%

**Table 2:** Distribution (geometric mean [ng/mL]) of urinary phthalate metabolites among the study participants.

Parent Phthalate	Abbreviation	Metabolites	Abbreviation	LOD (ng/mL)	%>LOD	CrudeGM (95% CI)	SG-adjusted GM (95% CI)
Di-n-butyl phthalate	DnBP	Monobutyl phthalate	MnBP	0.1	100.0	25.29 (22.09−28.96)	24.01 (20.54−28.07)
Diethyl phthalate	DEP	Monoethyl phthalate	MEP	0.5	95.7	6.33 (5.40−7.42)	6.01 (5.04−7.17)
Di-isobutyl phthalate	DiBP	Monoisobutyl phthalate	MiBP	0.1	100.0	11.18 (10.08−12.40)	10.62 (9.38−12.01)
		Mono2-ethyl-5-carboxypentyl phthalate	MECPP	0.1	97.1	5.19 (4.58−5.89)	4.93 (4.23−5.75)
Di-(2-ethylhexyl) phthalate	DEHP	Mono(2-ethyl)-hexyl phthalate	MEHP	0.1	100.0	1.36 (1.25−1.49)	1.29 (1.16−1.44)
		Mono(2-ethyl-5-oxohexyl) phthalate	MEOHP	0.1	97.6	3.00 (2.62−3.43)	2.84 (2.42−3.35)
		Mono(2-ethyl-5-hydroxyhexyl) phthalate	MEHHP	0.1	97.6	1.73 (1.55−1.94)	1.64 (1.43−1.89)
Dimethyl phthalate	DMP	Mononmethyl phthalate	MMP	2.0	26.4		
Di-n-octyl phthalate	DnOP	Mono(3-carboxypropyl) phthalate	MCPP	0.5	12.3		
Butyl benzyl phthalate	BBzP	Monobenzyl phthalate	MBzP	0.1	45.8		

Abbreviations: Geometric mean (GM), specific gravity (SG), limit of detection (LOD).

**Table 3:** Univariate analysis of urinary phthalate metabolites (geometric mean) based on the maternal demographic characteristics.

Maternal Characteristics	N = 210	MnBP ^a^	*p*	MEP ^a^	*p*	MiBP ^a^	*p*	∑DEHP ^a^	*p*
Maternal age (years)			0.328		0.130		0.021 *		0.322
20~24	23	29.25		4.58		12.92		42.83	
25~29	108	23.57		6.15		10.31		38.06	
30~34	54	25.01		7.77		11.90		43.45	
35~40	25	15.46		3.75		5.83		30.81	
Location			0.011 *		0.122		0.198		0.227
Urban	90	19.00		5.12		9.66		37.49	
Suburban	120	28.59		6.78		11.39		41.27	
Gestational weeks			0.213		0.018 *		0.537		0.298
First trimester	70	19.72		8.30		11.59		40.09	
Second trimester	70	27.77		6.09		10.69		43.93	
Third trimester	70	24.67		4.42		9.73		35.13	
Pre-pregnancy BMI			0.028 *		0.061		0.009 **		0.091
Underweight (<18.5)	21	52.30		9.01		19.17		57.45	
Normal (18.5–24.9)	154	23.55		5.49		9.83		36.61	
Overweight/Obese (≥25)	35	30.77		10.75		13.95		40.58	
Education			<0.01 **		0.205		0.001 **		0.126
High school or less	73	35.33		6.44		13.06		44.83	
College	56	24.12		7.26		12.27		40.51	
University or more	81	16.83		4.95		7.95		34.84	
Occupation			0.430		0.599		0.466		0.225
Production	10	27.20		6.12		12.68		26.69	
Service	31	18.88		4.81		10.18		34.24	
Management and technician	100	20.65		6.15		9.54		39.11	
Housework	53	29.80		7.17		12.33		46.39	
Others	16	45.09		4.46		12.24		43.64	

^a^ Molar sum of DEHP metabolites (MEHP, MEOHP, MEHHP, and MECPP) in nmol/L. GM: Geometric mean, in ng/mL. Using specific gravity (SG)-adjusted GM. * *p* < 0.05, ** *p* < 0.01.

**Table 4:** Univariate analysis of urinary phthalate metabolite concentrations based on the food group.

	Category	N	MnBP		MEP		MiBP		∑DEHP ^b^	
			GM ^a^	*p*	GM ^a^	*p*	GM ^a^	*p*	GM ^a^	*p*
Food Items	<1 time per day	113	25.95	0.291	6.35	0.513	10.90	0.656	40.54	0.662
	≥1 time per day	97	21.92		5.59		10.30		38.56	
Bottle water	YES	98	22.44	0.430	6.53	0.393	10.40	0.757	36.70	0.210
	NO	112	25.47		5.64		10.81		42.33	
Tofu	≤3 times per month	87	23.70	0.892	6.24	0.730	10.09	0.503	39.14	0.858
	≥4 times per month	113	24.23		5.86		11.00		39.95	
Pickle	<1 time per month	125	23.59	0.785	5.96	0.916	11.14	0.351	37.64	0.274
	≥1 time per month	85	24.66		6.08		9.88		42.73	
Edible seaweed	YES	125	21.62	0.146	8.00	0.008 **	12.45	0.035 *	42.82	0.515
	NO	85	21.81		4.94		9.51		37.55	
Milk	≤4 times per week	109	28.54	0.023 *	6.70	0.210	11.99	0.043 *	40.80	0.590
	≥5 times per week	101	19.89		5.34		9.30		38.37	
Yogurt	≤1 times per week	100	24.05	0.985	6.15	0.804	10.10	0.458	41.95	0.339
	≥2 times per week	110	23.98		5.88		11.10		37.62	
Pork	≤2 times per week	75	28.21	0.133	7.96	0.020 *	11.40	0.403	39.83	0.944
	>2 times per week	135	21.98		5.14		10.21		39.50	
Processed meat	≤3 times per month	168	24.90	0.364	5.61	0.120	10.51	0.745	38.38	0.264
	≥4 times per month	42	20.77		7.95		11.06		45.00	
Marine fish	<1 time per week	100	25.38	0.224	6.89	0.042*	11.52	0.139	42.14	0.229
	≥1 times per week	110	24.18		5.17		9.99		36.73	
Freshwater fish	<1 time per month	145	26.19	0.102	6.40	0.297	11.19	0.213	42.77	0.053
	≥1 times per month	65	19.76		5.22		9.44		33.36	
Egg	<1 time per day	68	25.82	0.531	6.22	0.792	11.48	0.394	45.33	0.676
	≥1 times per day	142	23.20		5.91		10.23		38.98	
Icecream	<1 time per month	177	23.52	0.543	5.96	0.841	10.57	0.886	39.33	0.764
	≥1 time per month	33	26.88		6.27		10.84		41.22	

^a^ Using specific gravity(SG)-adjusted concentrations in ng/mL. ^b^ Molar sum of di-(2-ethylhexyl) phthalate (DEHP) metabolites (MEHP, MEOHP, MEHHP, and MECPP) in nmol/L. Significant differences based on Student *t*-test, * *p* < 0.05, ** *p* < 0.01.We identified 9 predictors, including maternal age, location, gestational week, pre-pregnancy BMI, education level, and milk, pork, marine fish, and edible seaweed consumption, that were related to urinary phthalate metabolite concentrations in the univariate analyses. These were added to the adjusted model of multivariable linear regression analysis. In the multivariable models, lower educational level was still related with elevated urinary MnBP(*p* = 0.005) and MiBP concentrations (*p* = 0.006). Only edible seaweed remained a significant dietary predictor of urinary phthalate metabolite levels; participants who consumed edible seaweed on average had a 0.209-point increase in natural log transformed MEP levels (*p* = 0.005), a 0.191-point increase in natural log transformed MiBP levels (*p* = 0.012), and a 0.158-point increase in ΣDEHP concentrations (*p* =0.039), as compared to individuals that did not consume seaweed (Table 5).

**Table 5:** Multiple linear regression analysis of certain variables with urinary phthalate metabolite concentrations.

Characteristic	MnBP	*p*	MEP	*p*	MiBP	*p*	∑DEHP ^a^	*p*
Adjusted Model	ß (95% CI)		ß(95%CI)		ß (95% CI)		ß (95% CI)	
Maternal age	0.014 (−0.091~0.110)	0.851	0.112 (−0.029~0.199)	0.144	−0.019 (−0.090~0.069)	0.801	0.069 (−0.038~0.100)	0.378
Location	0.106 (−0.042~0.271)	0.150	0.107 (−0.047~0.309)	0.148	0.056 (−0.077~0.171)	0.457	0.139 (−0.008~0.208)	0.068
Gestational age	0.128 (−0.014~0.175)	0.096	−0.154 (−0.014~1.359)	0.055	−0.047 (−0.098~0.052)	0.543	−0.135 (−0.118~0.010)	0.096
Pre-pregnancy BMI	−0.104 (−0.294~0.047)	0.155	0.041 (−0.139~0.249)	0.575	−0.067 (−0.197~0.073)	0.365	−0.127 (−0.217~−0.017)	0.094
Education level	−0.223 (−0.227~−0.041)	0.005 **	−0.085 (−0.164~0.048)	0.283	−0.221 (−0.177~−0.029)	0.006 **	−0.093 (−0.104~0.026)	0.241
Milk consumption	−0.145 (−0.302~−0.006)	0.060	−0.047 (−0.229~0.121)	0.541	−0.115 (−0.214~0.031)	0.141	0.058 (−0.066~0.146)	0.482
Pork consumption	−0.071 (−0.232~0.082)	0.346	−0.090 (−0.285~0.071)	0.238	0.019 (−0.109~0.140)	0.804	0.055 (−0.069~0.146)	0.705
Marine fish consumption	0.006 (−0.150~0.161)	0.940	−0.092 (−0.283~0.070)	0.235	−0.003 (−0.126~0.121)	0.970	−0.012 (−0.115~0.099)	0.882
Edible seaweed consumption	0.130 (−0.016~0.286)	0.076	0.209 (0.073~0.416)	0.005 *	0.191 (0.035~0.274)	0.012 *	0.158 (0.006~0.213)	0.039 *

^a^ Molar sum of DEHP metabolites (MEHP, MEOHP, MEHHP, and MECPP) in nmol/L. * *p* < 0.05, ** *p* < 0.01.
